# Development of a Model for Dynamic Recrystallization Consistent with the Second Derivative Criterion

**DOI:** 10.3390/ma10111259

**Published:** 2017-11-02

**Authors:** Muhammad Imran, Markus Kühbach, Franz Roters, Markus Bambach

**Affiliations:** 1 Chair of Mechanical Design and Manufacturing, Brandenburg University of Technology Cottbus-Senftenberg, Konrad-Wachsmann-Allee 17, D-03046 Cottbus, Germany; bambach@b-tu.de; 2 Max-Planck-Institut für Eisenforschung GmbH, Max-Planck-Straße 1, D-40237 Düsseldorf, Germany; m.kuehbach@mpie.de (M.K.); f.roters@mpie.de (F.R.)

**Keywords:** dynamic recovery, recrystallization, second derivative criterion, alloy 800H

## Abstract

Dynamic recrystallization (DRX) processes are widely used in industrial hot working operations, not only to keep the forming forces low but also to control the microstructure and final properties of the workpiece. According to the second derivative criterion (SDC) by Poliak and Jonas, the onset of DRX can be detected from an inflection point in the strain-hardening rate as a function of flow stress. Various models are available that can predict the evolution of flow stress from incipient plastic flow up to steady-state deformation in the presence of DRX. Some of these models have been implemented into finite element codes and are widely used for the design of metal forming processes, but their consistency with the SDC has not been investigated. This work identifies three sources of inconsistencies that models for DRX may exhibit. For a consistent modeling of the DRX kinetics, a new strain-hardening model for the hardening stages *III* to *IV* is proposed and combined with consistent recrystallization kinetics. The model is devised in the Kocks-Mecking space based on characteristic transition in the strain-hardening rate. A linear variation of the transition and inflection points is observed for alloy 800H at all tested temperatures and strain rates. The comparison of experimental and model results shows that the model is able to follow the course of the strain-hardening rate very precisely, such that highly accurate flow stress predictions are obtained.

## 1. Introduction

Dynamic recrystallization (DRX) occurs in industrial hot working operations such as hot rolling and forging. DRX is often exploited to keep the forming forces low and to control the grain size, and thus the final product properties. Due to the large plastic deformation, complex geometry changes, and strong interactions between the evolution of the microstructure and the flow stress in hot working conditions, simulation methods such as the finite element method (FEM) are widely used for process design. For the description of the joint evolution of the microstructure and the flow stress, a plethora of phenomenological and physically-based models have been developed in the past.

When a microstructure-based flow stress model is implemented into a finite element formulation, it becomes a part of the constitutive model. It is widely accepted that a constitutive model must not violate the 2nd law of thermodynamics [1]. In computational mechanics, a check for thermodynamic consistency, i.e., compliance with the 2nd law of thermodynamics, has become a standard step in the development of constitutive models. In general, the constitutive models used in hot metal working belong to the class of elastic-viscoplastic models, for which thermodynamically consistent frameworks exist [2].

Although an elastic-viscoplastic model that contains a microstructure-based flow stress model such as models based on the equations proposed by Sandström and Lagneborg [3], Roberts and Ahlblom [4], Sellars [5], Laasraoui and Jonas [6], Sommitsch and Mitter [7], and Brown and Bammann [8] might formally be thermodynamically consistent, it might still violate conditions that have to be fulfilled at the onset of DRX. One such is compliance with the second derivative criterion of Poliak and Jonas [9], which states that at the onset of DRX, the strain-hardening rate as a function of the true stress develops an inflection point. This often-referred-to second derivative criterion (SDC) seems to be used predominantly [10,11,12,13] to detect the initiation of DRX from flow stress data. It has been confirmed with extensive experimental testing where the critical point has been detected as an inflection point in the strain-hardening rate as a function of stress [12,13,14,15,16,17]. However, as shown by Bambach [18,19], a flow stress model that incorporates DRX might suffer from inconsistencies with the SDC when it is not sufficiently many times differentiable at the critical point. When Johnson-Mehl-Avrami-Kolmogorov (JMAK) [20,21,22] kinetics are used to predict the DRX kinetics, inconsistencies occur when the Avrami exponent is less than or equal to three. As the second derivative criterion (SDC) by Poliak and Jonas was derived from principles of irreversible thermodynamics, a flow stress model which takes DRX into account should adhere to this criterion in order to be consistent with the experimental data and to assure the model to be sound in a thermodynamic sense.

A prerequisite for a flow stress model to comply with the SDC by Poliak and Jonas is that the predicted flow stress is sufficiently smooth, so that a continuous strain-hardening rate, as well as continuous first and second derivatives of the strain-hardening rate, can be computed. The demand for a continuous strain-hardening rate is also important in finite element formulations, since the computation of the tangent stiffness matrix in nonlinear FEA involves the strain-hardening rate. 

The current study analyses the necessary conditions which a flow stress model incorporating DRX needs to fulfill in order to be consistent with the Poliak and Jonas criterion. A brief summary of the analysis of different material models for the strain-hardening rate enables the identification of three types of potential inconsistencies with the SDC. A new model is proposed which eliminates all types of inconsistencies. The model is based on a single internal variable for strain-hardening, dynamic recovery, and DRX kinetics which combine Cahn [23] and Speich and Fisher [24] kinetics to describe the evolution of the interface between unrecrystallized and recrystallized grains. The comparison between the experiment and the model predictions substantiate the fulfillment of the SDC criterion. 

The paper is organized as follows: In Section 2, the mathematical prerequisites for enforcing thermodynamic consistency with the SDC are derived and the corresponding three types of inconsistencies identified. In Section 3, an overview of the investigated material, the experimental setup, and the deformation conditions are presented. Section 4 presents the hot deformation flow curve and corresponding strain-hardening rate evolution. Post-deformation microstructural behavior and the relationship between characteristic points on the strain-hardening rate curves and the Zener-Hollomon-parameter is also presented in Section 4. Based on consistency with the SDC, a new single-internal-variable model is developed in Section 5 and validated with the experimental results in Section 6.

## 2. Criteria for Consistency with the SDC

### 2.1. Mathematical Implications of the SDC

The second derivative criterion by Poliak and Jonas [9] states that at the onset of DRX, the second derivative of the strain-hardening rate *θ* = *∂σ*/*∂ε* with respect to flow stress *σ* vanishes:

(1) $$\kappa \left(\sigma \right)=\frac{\partial }{\partial \sigma }\left(-\frac{\partial \theta_{C}}{\partial \sigma }\right)=0$$

Therefore, computing the second derivative of the strain-hardening rate is necessary in order to determine the inflection point in the strain-hardening rate *θ*, and thus, the critical conditions for the initiation of DRX. Numerically, this requires three-times continuous differentiability of the stress-strain curve [18,19]. The SDC can be rewritten in the following form:

(2) $$\frac{\partial^{2}\theta }{\partial \sigma^{2}}=\frac{{\left(\theta^{\prime }\right)}^{2}-\theta^{\prime \prime }\cdot \theta }{\theta^{3}}\;\neq 0$$

in which ()’ denotes differentiation with respect to strain. To get consistent results, it is also required that

(3) $$\underset{\varepsilon \to \varepsilon_{c}^{-}}{\lim}\kappa \left(\varepsilon \right)=\underset{\varepsilon \to \varepsilon_{c}^{+}}{\lim}\kappa \left(\varepsilon \right)$$

“+” and “−” indicate ε > ε_c_ and ε < ε_c_, respectively. A detailed analysis of the implications for DRX kinetics following from Equations (2) and (3) given in [18,19] shows that, for most models, independent of the mixture law, *X_DRX_* and its first three derivatives with respect to strain need to be equal to zero at the onset of DRX. In short, the violation of Equations (2) and (3) leads to an inconsistent model and should therefore be avoided.

Besides limited differentiability, two additional inconsistencies can occur in models, which will be discussed in proceeding section.

### 2.2. Inconsistencies with the SDC

**Figure 1:**
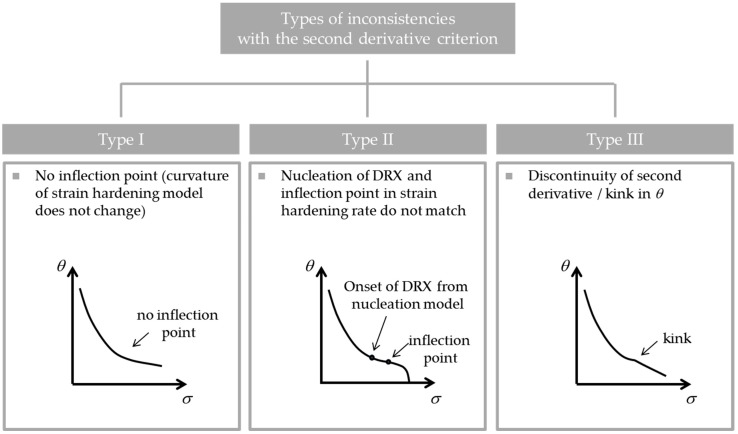
Schematic representation of three types of inconsistencies with the second derivative criterion (SDC) [25].

Three inconsistencies with the SDC can be identified, as illustrated in Figure 1.

(i) The hardening model does not produce an inflection point in the strain-hardening rate.
(ii) The point at which the criterion identifies the location of the point of inflection does not match the actual point of inflection in the experimentally measured strain-hardening rate.
(iii) The derivatives of the strain-hardening rate are discontinuous.

#### 2.2.1. Type *I* Inconsistency

If the part of the model that is responsible for strain-hardening and softening of the parent polycrystal before the initiation of DRX does not show a point of inflection in the strain-hardening rate, Equation (2) can obviously not be fulfilled. An excellent example is the usage of a Kocks-Mecking-type equation for the strain-hardening rate [26], which is a linear function in a Kocks-Mecking plot:

(4) $$\theta =\theta_{0}\cdot \left(1-\frac{\sigma }{\sigma_{0}}\right)$$

Most single parameter models suffer from this inconsistency as at least a second internal variable is needed to model stage IV hardening, cf. [27].

#### 2.2.2. Type *II* Inconsistency

Another type of inconsistency is observed when the nucleation criterion is inconsistent with the inflection point in the strain-hardening model. A common nucleation model for the onset of DRX was proposed by Roberts and Ahlblom [4] based on the classical nucleation theory. If the strain-hardening model shows an inflection point which does not coincide with the point where the critical dislocation density is reached according to the nucleation model, the model should be considered inconsistent with the SDC. This type of inconsistency is likely to occur when strain-hardening models with a single internal variable are combined with the nucleation criterion by Roberts and Ahlblom [4] or when semi-empirical models are used, which express the critical strain for DRX as a function of the Zener-Hollomon-parameter. In both cases, there is no mechanism which ensures the prediction of the critical point by the nucleation sub-model to match with the prediction of its location by the strain-hardening model, i.e., the occurrence of an inflection point in the strain-hardening rate does not trigger the “nucleation criterion”.

#### 2.2.3. Type *III* Inconsistency

Usually, DRX models make use of a mixture law combining the flow stress in the recrystallized and non-recrystallized volume fractions. In this case, the DRX kinetics is switched on at the critical point creating the possibility for a discontinuity in the derivative of *θ*. Most models use an Avrami-type approach to model the evolution of the recrystallized volume fraction. Bambach [19] analyzed the importance of the Avrami exponent for the fulfillment of Equation (3) and showed that the Avrami kinetics must exceed a value of 3 to be consistent. Therefore, it requires the utilization of generalized Avrami kinetics instead of the conventional set of conditions.

### 2.3. Inconsistencies in Strain-Hardening Models for Hot Working

**Figure 2:**
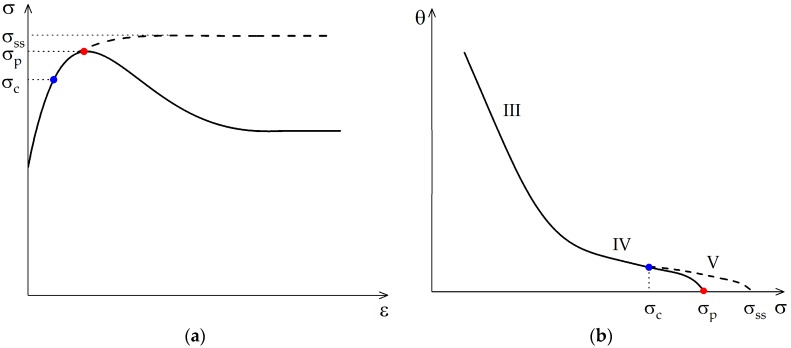
In (**a**) it is shown schematically the evolution of flow stress with (true) strain in the case of a single peak due to dynamic recrystallization (DRX). The dashed line displays the theoretical case of a solely dynamically recovering microstructure, i.e., DRV whereas the solid line includes DRX effects. Sub-figure (**b**) details the corresponding strain-hardening rate evolution in Poliak Jonas space which shows an inflection point (blue dot) and peak stress point (red dot).

The strain-hardening rate evolution as a function of stress is usually divided into three stages (*III*, *IV* and *V*, see dashed line in Figure 2). Stage *III* is characterized by a linear decrease of the strain-hardening rate with increasing stress. It is controlled by cross-slip of screw dislocations and can be modeled well with Kocks-Mecking-type approaches [26,28]. These compute the evolution of an effective dislocation density as a single internal variable. During stage *IV*, though, this constant reduction of the strain-hardening rate levels out to a lower but, again, often constant rate. The critical stress (inflection) marks the transition to stage *V*, which is characterized by an increasingly fast drop of the strain-hardening rate towards zero.

Flow stress models for hot working can be divided into two general categories: phenomenological [5,29,30] and physically-based models. Phenomenological models employ empirical functions that have shown the ability to reproduce the experimentally observed behavior. They can be subdivided further into purely empirical and semi-empirical models. Semi-empirical models express characteristic points of the flow curve such as the peak strain and stress as a function of the Zener-Hollomon-parameter. By contrast, empirical models are simply strain-rate and temperature dependent.

Physically-based models make use of thermodynamically meaningful internal variables such as dislocation densities. They can be subdivided into models which consider DRX via an explicit spatial resolution of the microstructure [14,31,32] and those without [4,6,7,8,33].

Despite these distinctions, flow stress models typically include 4 different parts or sub-models, respectively [18]:

(i) Model for strain-hardening and dynamic recovery
(ii) Nucleation criterion for DRX
(iii) Function describing the dynamically recrystallized volume fraction as a function of strain or time
(iv) Rule of mixture to determine the macroscopic flow stress when recrystallized and non-recrystallized grains coexist

The consistency of a model should be determined by all 4 parts. Bambach [25] analyzed the typical models incorporating DRX and the consistency of the models with respect to the SDC. In general, the first inconsistency occurs if the hardening model uses only stage *III*, stage *IV*, or a combination of both stages, e.g., [15,34,35,36]. The second inconsistency usually is a direct consequence of the first type. Not producing an inflection but still modeling DRX leads to a pseudo stage *V* that solely stems from DRX, since DRX leads to an increasing drop of the strain-hardening rate [7,15,29,35]. The third type of inconsistency is a consequence of the Avrami exponent. If *n* denotes the Avrami exponent, then the first *n* − 1 derivatives of the flow stress need to be discontinuity-free, which mean for exponent ≤ 3 the strain-hardening rate and, subsequently its derivatives will show discontinuity. For an exponent > 3, consistency with the SDC can be achieved [18]. None of the models from the literature that were analyzed with regard to their consistency with the SDC is fully consistent. In the subsequent section, a phenomenological model is proposed to fulfil the criteria to be consistent with the SDC. 

## 3. Material and Experimental Methodology

In the present study, the investigated material is X10NiCrAlTi2032, an austenitic steel, also known as Alloy 800H. Its principal alloying elements are nickel and chromium, as Table 1 details. DRX in Alloy 800H was analyzed in various previous studies [12,37,38], so that the results obtained in this work may be compared to other references.

The alloy has an excellent heat resistance, ability to withstand the oxidation at high temperature, and reasonable corrosion resistance. The material for experimental and microstructure investigations was commercially acquired. The delivered material was hot-rolled, solution annealed, and centerless ground.

Hot compression cylindrical specimens of 5 mm in diameter and 8 mm in height were extracted from the round bar material. The microstructure of the samples in undeformed condition and after hot deformation testing was analyzed using optical microscopy Leica DM4000M. The measurement sections expose the microstructure of the sample core both perpendicular and parallel to the deformation axis. The preparation comprised wet-metallographic grinding with SiC paper, successive diamond suspension mechanical polishing with 3 µm and 1 µm and contrasting using V2A etchant at 50 °C for 30 s.

The hot deformation testing was carried out in a Dilatometer DIL805 equipped with a linear variable differential transducer (LVDT). The heat transfer from the hot specimen to the supporting surfaces of the compression punch leads to an undesired temperature gradient and, consequently, an inhomogeneous deformation of the specimen. To reduce such inhomogeneity, molybdenum discs with a thickness of 1 mm were placed between the specimen and the compression punch, which was also heated by the induction coils. Aqueous graphite was deployed between the sliding surfaces of the specimen and molybdenum disc to minimize frictional effects. Hot deformation tests were performed for 7 temperatures and 3 strain rates. The specimens were heated using induction coils up to the deformation temperature at a heating rate of 10 K/s and held for 5 min to ensure complete heating of the specimen. The specimens were deformed under a vacuum of 10^−4^ bar. Immediately thereafter, the microstructure was frozen as fast as technically possible via exposure to Helium gas resulting in a cooling rate of 200 K/s.

## 4. Experimental Results

### 4.1. Microstructure Characterization

**Figure 3:**
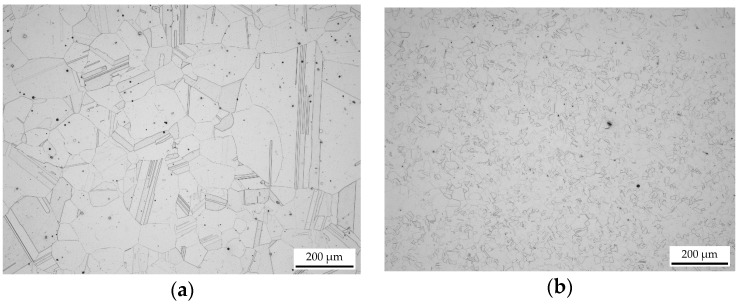
Microstructure of Alloy 800H in the (**a**) as-delivered state; and (**b**) after compression at 1100 °C with a strain rate of 1 s^−1^ to a total strain of 80%. The compression axis runs parallel to the vertical image edge.

Figure 3a shows the microstructure in the as-delivered state. The optical microscopy investigations evidence a heavily-twinned annealing microstructure with an ASTM E112 standard grain size between 2.5 and 3.5. The microscopic investigations of the deformed specimens show a symmetrical geometry of the heat-affected zone at the upper and lower region to the central axis of the sample. The microstructure are partially recrystallized at low temperatures (900–1050 °C) and fully recrystallized at higher temperatures (1100–1200 °C). Figure 3b depicts the fully dynamically recrystallized microstructure after compression at 1100 °C and a strain rate of 1 s^−1^. The microstructure shows substantial evidence of annealing twinning. Consequently, many triangular grain cross-sections and other non-equilibrium projected grain boundary contact angles at the triple junctions are evidenced.

### 4.2. Hot Deformation Behavior

**Figure 4:**
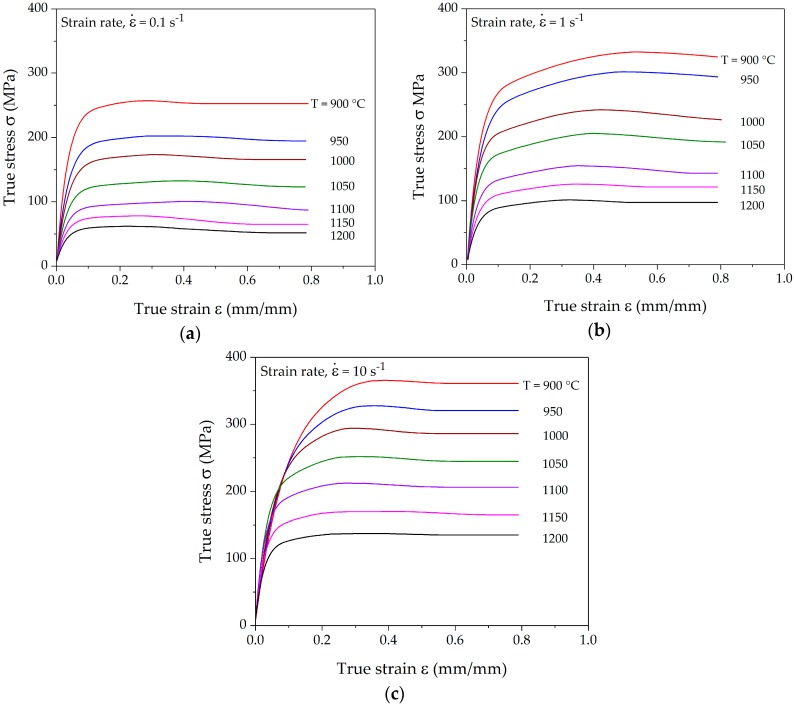
Representative true stress-strain curves for hot compression at different temperatures and strain rates of (**a**) 0.1 s^−1^; (**b**) 1 s^−1^; and (**c**) 10 s^−1^.

Figure 4 displays the deformation characteristics in the form of true stress-true strain curves for the temperature range between 900–1200 °C in 50 °C steps and strain rates of 0.1, 1, and 10 s^−1^. Polynomials of degrees in the range of 8–11 were fitted to obtain smooth flow curves and strain-hardening rates, as proposed by Poliak and Jonas [9] and Jonas et al. [34].

The flow curves show the typical evidence of dynamic recrystallization behavior via their distinct peak stress. The steady state is reached much earlier at lower strain rates as compared to higher strain rates. In the case of low strain rates, a relatively sluggish hardening rate is observed as a consequence of low plastic strain energy. Similar behavior is also reported in the experimental observation by Cao et al. [39].

**Figure 5:**
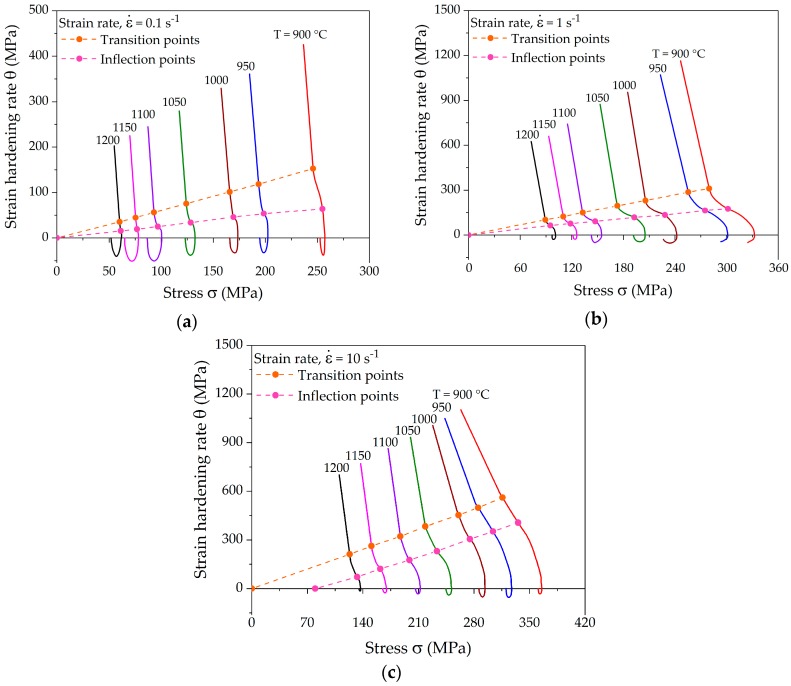
Characteristic strain-hardening rate curves at different temperatures and strain rates of (**a**) 0.1 s^−1^; (**b**) 1 s^−1^; and (**c**) 10 s^−1^ showing a linear shift of the transition and inflection points.

A typical strain-hardening rate curve contains three stages with distinct separation points between the different stages. Figure 5 shows the experimentally determined strain-hardening rates in Kocks-Mecking plots for various deformation conditions corresponding to the flow curves presented in Figure 4. All the hardening curves show the distinct transition and inflection points. The strain-hardening rate in stage *III* decreases rapidly in a linear manner. The slope of stage *IV* decreases marginally as compared to stage *III* in the case of the lowest strain rate. Such a small slope can be attributed to a high dynamic recovery rate due to slow deformation. Stage *IV* is more distinct at higher strain rates, where the slope of the hardening rate decreases considerably. After the onset of DRX, the strain-hardening rate decreases much faster towards the peak stress, at which *θ* reaches 0 for lower strain rates, as shown in Figure 5a. It can be observed that both the transition and inflection points fall onto a line in the Kocks-Mecking plots at a constant strain rate. The dependence of these points on the Zener-Holloman-parameter is analyzed in the subsequent section.

### 4.3. Dependence of Characteristic Points on the Zener-Hollomon-Parameter

The true stress-strain curves provide a qualitative measure of microstructure evolution as a function of temperatures and strain rates. The influence of deformation conditions on strain hardening rates can be quantified with characteristic stresses such as (i) the transition between stage *III* and stage *IV* (*σ_III_*); (ii) the inflection point at the onset of dynamic recrystallization (*σ_c_*); and (iii) the point at peak stress (*σ_p_*). *σ_III_* and *σ_c_* are distinctly marked in the experimental strain-hardening rate plots in Figure 5. In hot metal forming, it is expedient to correlate the characteristic points with the so called Zener-Hollomon-parameter (*Z*), also termed the temperature-compensated strain rate. The dependence of these points on *Z* can be expressed by an Arrhenius equation as detailed with Equation (5):

(5) $$Z=\dot{\varepsilon }exp\left(\frac{Q_{w}}{RT}\right)=A{\left[\sin h\left(\alpha \sigma \right)\right]}^{n}$$

in which *T* and $\overset{´}{\dot{\varepsilon }}$ denote the deformation temperature and strain rate, respectively, *R* the molar gas constant, and *A*, *α*, and *n* are material constants. *Q_w_* (365 ± 15 kJ∙mol^−1^) is the activation energy for hot deformation. 

The first part of Equation (5) correlates *Z* with deformation conditions, whereas the term [sinh(*ασ*)] shows the dependency of the characteristic stresses *σ* on *Z*, i.e., *σ_III_*, *σ_c_*, or *σ_p_*, respectively. The characteristic stress points need to be determined from the strain-hardening rate. Table 2 details the results of fitting parameters (*A*, *α,* and n) for Equation (5).

**Figure 6:**
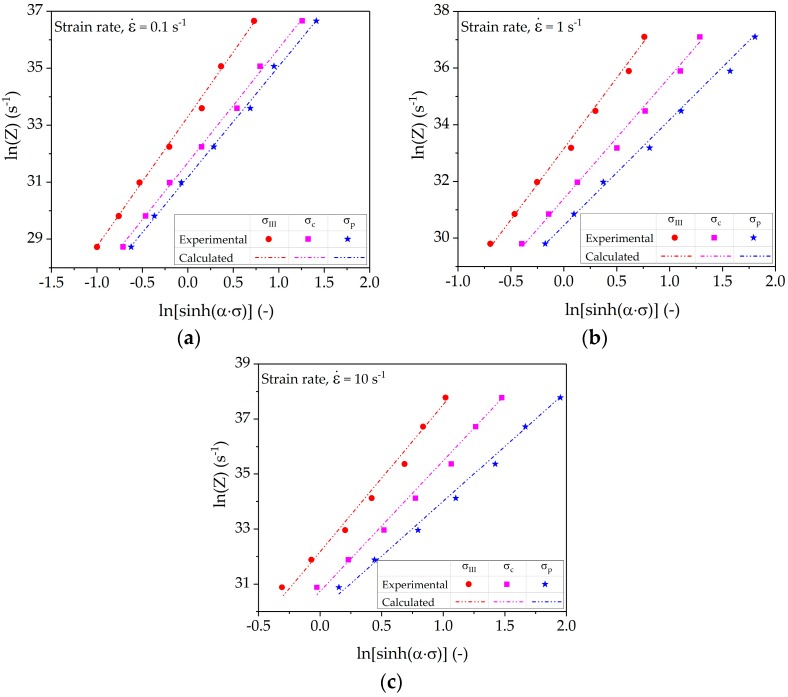
Characteristics points (*σ_III_*, *σ_c_*, or *σ_p_*) dependency on Zener-Hollomon-parameter at strain rates of (**a**) 0.1 s^−1^; (**b**) 1 s^−1^; and (**c**) 10 s^−1^.

Figure 5 shows a linear shift of characteristic points at different temperatures at fixed strain rate. The dependence of the linear shift of *σ_III_, σ_c_*, and *σ_p_* on *Z* is shown in Figure 6. The comparison of experimental *Z* values determined using deformation parameters and calculated *Z* values determined using characteristic stress values is also presented. The dependence of *σ_III_, σ_c_*, or *σ_p_* on *Z* was determined using nonlinear regression analysis of Equation (5). The results depict the fact that the linear shift of the characteristic points shows a linear dependence on ln *Z*. Moreover, the slope of the regression lines decreases with increasing strain rate. The statistical analysis of the data comparison shows an R-squared value higher than 99.5% for the fitted equations, proving the high accuracy of the linear function.

## 5. Thermodynamically Consistent Strain-Hardening Model for Hot Working

In the following, a new model consistent with the SDC is proposed. To achieve consistency with the SDC, the following rules have to be fulfilled:

(i) The hardening model needs to take all three hardening stages into account.
(ii) The criterion for the initiation of DRX has to match the inflection in the strain-hardening rate.
(iii) The first three derivatives of the flow stress function with respect to the strain have to be continuous. The function describing the DRX kinetics and its first three derivatives have to vanish at the critical point.
(iv) In the case of an Avrami-type approach, the Avrami exponent has to be greater than 3.

### 5.1. Strain-Hardening Model

The new strain-hardening model is devised based on the observations of the course of the strain-hardening rates in the *θ*-*σ*-space. A schematic overview of the different hardening stages to be modeled is presented in Figure 2 showing transition from linear hardening stage *III* to nonlinear hardening stage *V* and the inflection point at the onset of dynamic recrystallization.

To include all stages *III*–*V* in the hardening model, a model composed of three different functions adapted to the stages *III*–*V* is proposed. Switching between the parts of the model is accomplished using sigmoidal functions. The switching points are modeled based on the linear course of these points in *θ*-*σ*-space observed from Figure 5. The linear decrease in stage *III* hardening rate is modeled using the Bergström model [40] and the simplified formulation can be written as:

(6) $$\theta_{III}=b_{III}-m_{III}\sigma$$

where *b_III_* and *m_III_* are the intercept and slope of stage *III*, respectively.

After the transition point (*σ_III_*), the decrease in strain-hardening rate decelerates due to the increased dislocation annihilation rate. Thus, the gradient of *θ* in stage *IV* is much smaller than in stage *III* (*m_IV_* < *m_III_*) because of sharpening of the cell walls. A linear decrease in stage *IV* around the critical point for DRX initiation is assumed:

(7) $$\theta_{IV}=b_{IV}-m_{IV}\sigma$$

The slope of stages *III* and *IV* in Equations (6) and (7) is calculated by determining the relationship between the slope and the Zener-Hollomon-parameter. For such formulation, the evolution equation for dislocation density according to Bergström [40] is considered, which reads:

(8) $$\frac{d\rho }{d\varepsilon }=U\left(\rho \right)-\Omega \left(\dot{\varepsilon },\;T\right)\rho$$

with

(9) $$U\left(\rho \right)=U_{0}\sqrt{\rho }$$

and

(10) $$\Omega \left(\dot{\varepsilon },\;T\right)=\Omega_{0}+C_{1}exp\left(-\frac{mQ_{w}}{RT}\right){\dot{\varepsilon }}^{-m}=\Omega_{0}+C_{1}Z^{-\alpha }$$

Since

(11) $$\sigma =C_{2}\sqrt{\rho };\;\theta =\frac{d\sigma }{d\varepsilon }=C_{2}\frac{d\rho /d\varepsilon }{2\sqrt{\rho }}$$

(12) $$\theta =\frac{C_{2}}{2\sqrt{\rho }}\left(U_{0}\sqrt{\rho }-\left(\Omega_{0}+C_{1}Z^{-\alpha }\right)\rho \right)$$

Comparing Equations (12) and (6) or (7) and combining the constants, the formulation reads:

(13) $$\ln(m_{III,\;IV})=C-\alpha \cdot ln\left(Z\right)$$

**Figure 7:**
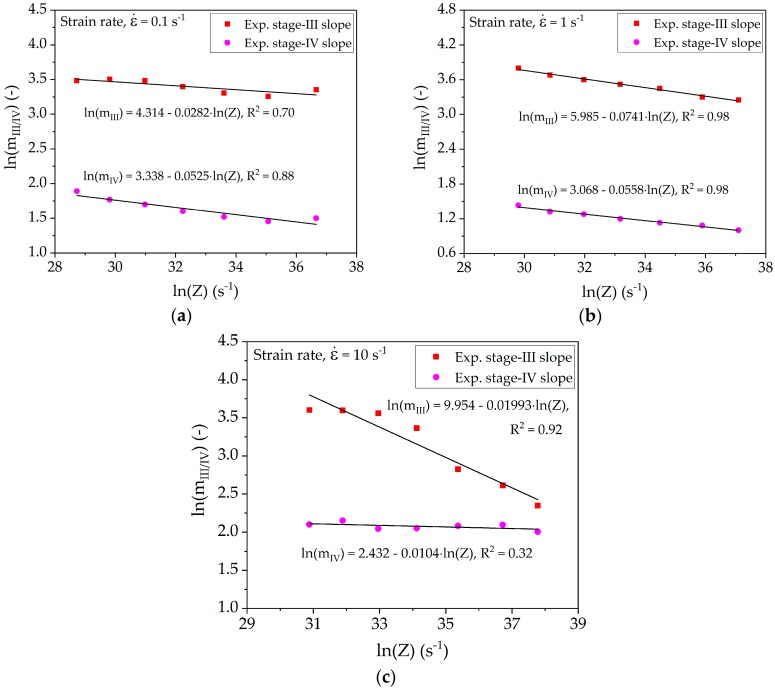
Relationship between slope of stage *III* (*m_III_*), stage *IV* (*m_IV_*), and Zener-Hollomon-parameter at strain rate of (**a**) 0.1 s^−1^; (**b**) 1 s^−1^; and (**c**) 10 s^−1^.

Equation (13) describes the dependence of slope of stage *III* and *IV* on the Zener-Hollomon-parameter. Figure 7 represents the plots of the slopes determined from experimental strain-hardening rates together with model fitting according to Equation (13).

Table 3 shows the average values of parameters *b_III_* and *b_IV_* used for the model calibration. The Bergström model does not present any formulation for the intercepts and the systematic dependence of these parameters on the deformation conditions is also not part of this study. Further work should include a formulation for *b_III_* and *b_IV_*.

Finally, considering the nonlinear decrease in the strain-hardening rate, the diffusion-driven climb of immobile dislocations in stage *V* is modeled as follows:

(14) $$\theta_{V}=-C\left(\sigma -\sigma_{c}\right)$$

The complete model in the form of a piecewise-defined function can be written as:

(15) $$\theta \left(\sigma \right)=\{\begin{array}{ccc} \theta_{III} & & \sigma \leq \sigma_{III}\\ h\left(x,\;x_{1},x_{2}\right) & =\left(\theta_{IV}-\theta_{III}\right)H_{1}\left(x\right), & \sigma_{III}<\sigma <\sigma_{c}\\ \theta_{V} & & \sigma \geq \sigma_{c} \end{array}$$

The transition from stage *III* to *IV* is achieved through a transition function *h*(*x*, *x*_1_, *x*_2_), where *x* is place holder for *σ, x*_1_ and *x*_2_ accounts for the beginning and end of the deviation from linear slop, respectively. The transition function provides continuous differentiability to the second order derivative of *θ*. The incorporated boundary conditions are as follows:

(16) $$h\left(x_{1}\right)=\theta_{III}\left(x_{1}\right),\;h^{\prime }\left(x_{1}\right)=m_{1},\;h^{\prime \prime }\left(x_{1}\right)=0\;h\left(x_{2}\right)=\theta_{IV}\left(x_{1}\right),\;h^{\prime }\left(x_{2}\right)=m_{2},\;h^{\prime \prime }\left(x_{2}\right)=0$$

To achieve a smooth transition, the function *H*_1_(*x*) is selected as follow:

(17) $$H_{1}\left(x\right)=\frac{1}{2}+\frac{1}{2}\tanh\left(cx\right)$$

### 5.2. Nucleation Criterion

The onset of DRX is explicitly modeled using an asinh-dependence (cf. Equation (5)). Therefore, the rule (ii) has to be considered carefully. Consistency with the SDC is achieved because the slope of *H*_1_(*x*) is positive as per definition and approaches zero at the critical stress while the term *σ* > *σ_c_* has a negative curvature that approaches zero at the critical stress.

### 5.3. Flow Stress Model

The flow stress is obtained by transforming the strain-hardening rate into stress-strain space. Up to a constant offset in hardening rate, the transformation corresponds to a Legendre transform. 

To combine the strain-hardening model with the recrystallization kinetics, a linear rule of mixture is used:

(18) $$\sigma \left(\varepsilon \right)=\;\sigma_{0}+\sigma_{DRV}-\Theta \left(\varepsilon -\varepsilon_{c}\right)\cdot \left(\sigma_{DRV}^{ss}-\sigma_{ss}\right)\cdot X_{DRX}$$

Therein, *σ_0_* denotes the initial flow stress, Θ(*ε* − *ε_c_*) the Heaviside step function, $\sigma_{DRV}^{ss}$ the steady state flow stress of the hardening model, $\sigma_{ss}$ the steady state flow stress, and *X_DRX_* the recrystallized volume fraction.

### 5.4. Dynamic Recrystallization Model

To model the DRX kinetics, two effects need to be taken into account, namely the continuous generation of recrystallization nuclei and saturation of nucleation sites (site saturation). In the case of continuous nucleation, it is assumed that the nuclei grow spherically from randomly distributed locations within the microstructure. Cahn analyzed the kinetics of volume transformation when nucleation takes place from randomly distributed locations on grain boundary faces, and junctions (grain boundary edges and corners) under the assumption that the nuclei grow spherically [23]. He derived transformation kinetics with an exponent of 4, which happens to be consistent with the SDC. Since most metallographic investigations including those of Roberts and Ahlblom [4] make no explicit quantitative assessment of the significance of nucleation at the junctions it is assumed that randomly distributed nucleation on the grain boundary faces is decisive. In this case, the evolution of recrystallized volume fraction can be calculated as [23]:

(19) $$X=1-\exp\left[-b_{s}^{-1/3}f\left(a_{s}\right)\right]$$

where

(20) $$f\left(a_{s}\right)=a_{s}\int_{0}^{1}\left[1-\exp\left(-\pi a_{s}^{3}\left\{\frac{1-x^{3}}{3}-x^{2}\left(1-x\right)\right\}\right)\right]dx$$

with $a_{s}={\left(I_{s}G^{2}\right)}^{1/3}t;$ $b_{s}=\frac{I_{s}}{8S^{3}G}$ .

In Equations (19) and (20), *X* is the recrystallized volume fraction at time *t*, *I_s_* the specific surface nucleation rate, *G* the radial growth velocity, and *S* the grain surface area per unit volume. 

Roberts and Ahlblom [4] suggested that Cahn kinetics is only valid up to the point when all initial grain boundary face area was swept by the nuclei, i.e., site saturation, and then DRX should be modeled by an interfacial approach. Therefore, in the present work, modeling of the DRX kinetics is achieved by starting with the model proposed by Cahn for transformation originating at former grain boundaries. The kinetics then transition into a model proposed by Speich and Fisher [24], which assumes nucleation at the interface between recrystallized and deformed grains after all initially available nucleation sites have been occupied. The empirical relation is given as

(21) $${\dot{X}}_{DRX}=\xi \cdot A_{I}$$

*ξ* is the average velocity of the interface as it sweeps through the non-recrystallized volume, and *A_I_* = *X*·(1 − *X*) is the interface area between the recrystallized and non-recrystallized volume. This approach takes the saturation of sites into account but is not suited to model the beginning of DRX, since *X_DRX_* > 0 is needed as an initial value. It is hence suggested to combine the models by Cahn with the one by Speich and Fisher which reads in ODE form as follows:

(22) $$\dot{X}\left(t\right)=\left(1-X\left(t\right)\right)\left[\left(1-H\left(t_{ss},\;t_{s}\right)\right)\frac{4\pi }{3}I_{s}SG^{3}t^{3}+H\left(t_{ss},\;t_{s}\right)AX\left(t\right)\right]$$

Therein *H*(*t_ss_*, *t_s_*) is a switch function between the time at site saturation (*t_ss_*) and *t_s_* the duration of the transition from nucleation at pre-existing grain boundaries to nucleation at interfaces, i.e., *H* = 0 at *t* = *t_s_* and *H* = 1 at *t* = *t_ss_* + *t_s_*.

## 6. Model Validation and Discussion

**Figure 8:**
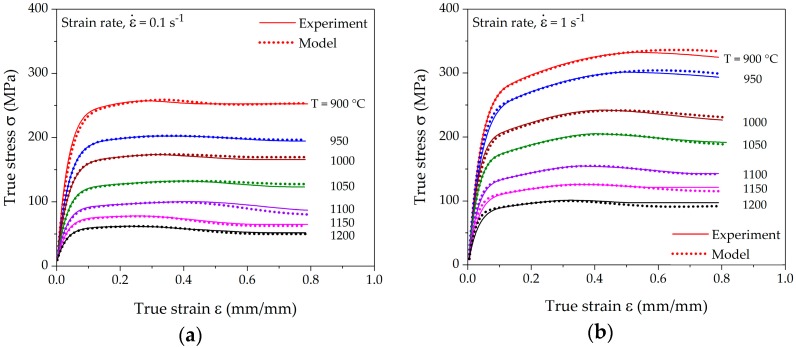

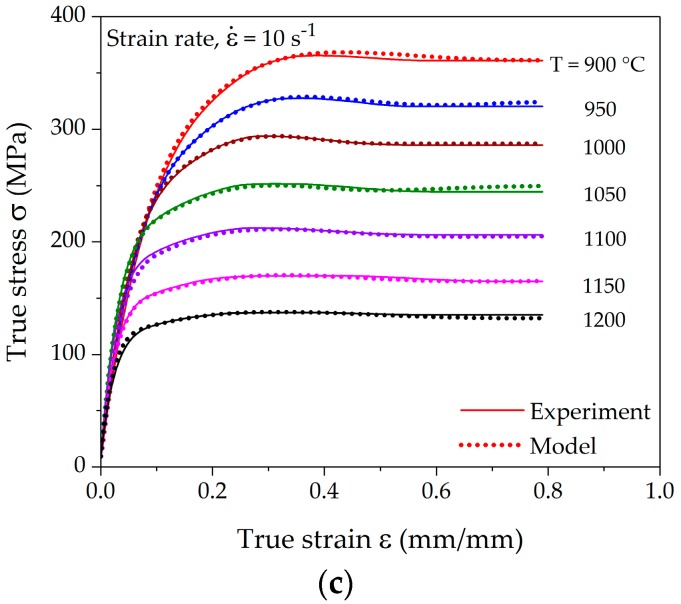
Fit of the computed flow curves with measured data from hot compression tests at different temperatures and strain rates of (**a**) 0.1 s^−1^; (**b**) 1 s^−1^; and (**c**) 10 s^−1^.

**Figure 9:**
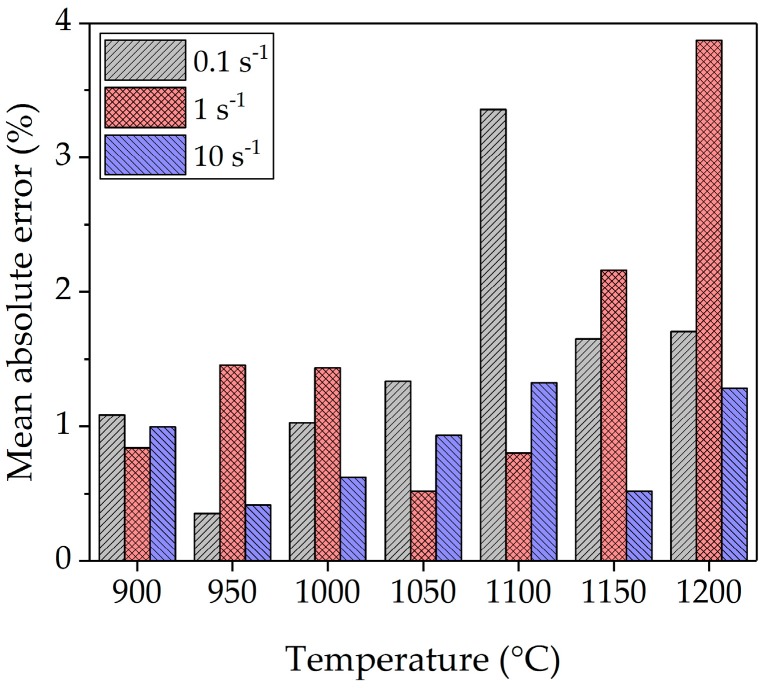
Mean absolute percentage error of the new model.

The model was fitted and evaluated for all experimentally measured strain-hardening rate curves and transformed to σ-ε-space from θ-σ-space to determine the accuracy of the model. Figure 8 displays the comparison of the experimental and modeled flow curves at different deformation conditions. The experimental flow curves were determined by individually fitting a polynomial of the order 8–11. However, such interpolation may yield multiple critical points. In such case, a more sophisticated interpolation technique, thin plate spline (TPS), can be utilized as proposed by Lohmar and Bambach [41]. The accuracy of the interpolation was quantified using the mean absolute percentage error (MAPE).

(23) $$MAPE=\frac{100}{n}\overset{n}{\underset{i=1}{\sum }}\left|\frac{y_{i}-{\hat{y}}_{i}}{y_{i}}\right|$$

where *n* is the number of data points supporting the flow curve, $y_{i}$ the actual and ${\hat{y}}_{i}$ the predicted value. The statistical results in Figure 9 show acceptable deviations with a mean absolute percentage error of less than 4% for all fitted curves.

**Figure 10:**
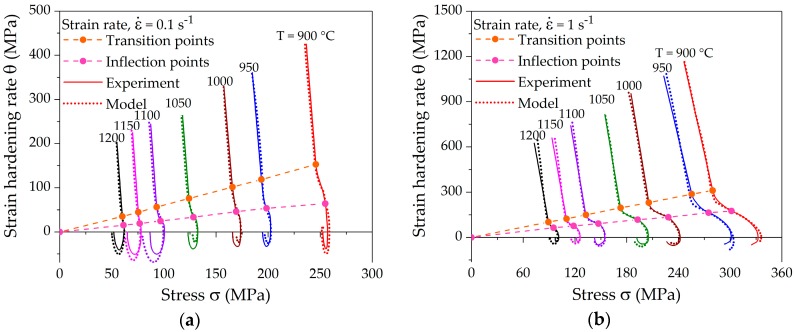

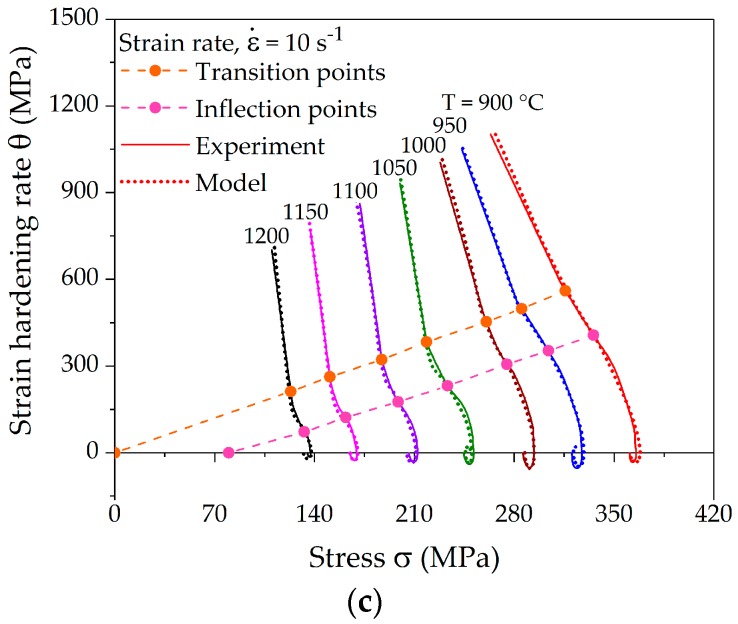
Fit of the computed strain-hardening rate curves with measured data from hot compression tests at different temperatures and strain rates of (**a**) 0.1 s^−1^; (**b**) 1 s^−1^; and (**c**) 10 s^−1^.

The comparison of computed and measured strain-hardening rates is presented in Figure 10. The computation of strain-hardening rates from the new model yields the distinct characteristic points, which coincide with the experimental values. The fitting results show that the model is able to precisely follow all hardening stages *III*–*V*, as well as the course up to the steady state.

Although the validation results of the models show a good agreement with the experimental data, it is noteworthy to describe some possible limitations of the model. At the moment, the model is only validated for the data which have been used to calibrate the model. The developed model is still a phenomenological model relying on experimentally determined characteristic points; the extrapolation behavior of model beyond the measured data sets is not studied. The model uses a large number of parameters; parameter correlations should be analyzed as performed in [42].

## 7. Conclusions

1. We identified three inconsistencies of flow stress models for dynamically recrystallizing microstructures with the second derivative criterion (SDC):
  (i) The hardening model does not produce an inflection point in the strain-hardening rate.
  (ii) The predicted point of inflection does not match the inflection in the strain-hardening rate.
  (iii) The derivatives of the strain-hardening rate are discontinuous.

According to these findings, none of the models from the literature that were analyzed with regard to consistency with the SDC are fully consistent. Inconsistency with the SDC leads to kinks or discontinuities in the strain-hardening rate and its derivatives, which can lead to divergence in FE simulations. Based on these findings

2. A new, single-internal-variable model consistent with the SDC was proposed. The model is based on the course of the strain-hardening rate as a function of stress, which is modeled using three distinct model functions. The transition point between stages *III* and *IV* and the critical stress for DRX are modeled as linear functions in the Kocks-Mecking space.
3. The model is transformed into stress-strain space using a modified Legendre transform. It is combined with DRX volume transformation kinetic models by Cahn [23] and Speich and Fisher [24], thus yielding DRX kinetics consistent with the SDC.
4. The comparison of the modeling results with the experimental data shows a reasonable accuracy in the Kocks-Mecking plots and the flow stress.

## Figures and Tables

**Table 1:** Chemical composition of Alloy 800H.

Element	Ni	Cr	Mn	Si	Ti	Al	C, Co, Cu, S	Fe
wt. %	30.25	20.51	0.69	0.50	0.36	0.26	<0.09	Bal.

**Table 2:** Parameters of constitutive equation for strain-hardening rate characteristic points.

Characteristic Points	Strain Rate, s^−1^	*A*, s^−1^	*α*, MPa^−1^	*n*
σ_III_	0.1	2.9∙10^14^	0.0060	4.55
	1.0	2.50∙10^14^	0.0053	5.00
	10	9.4∙10^13^	0.0055	5.35
σ_c_	0.1	5.8∙10^13^	0.0077	4.01
	1.0	4.3∙10^13^	0.0065	4.32
	10	2.2∙10^13^	0.0064	4.75
σ_p_	0.1	3.4∙10^13^	0.0082	3.89
	1.0	2.1∙10^13^	0.0072	3.72
	10	1.1∙10^13^	0.0072	3.98

**Table 3:** Intercept values for stage *III* and *IV*.

Parameter	Strain Rate, s^−1^		
	0.1	1.0	10
*b_III_*	4042.44	5867.55	5408.66
*b_IV_*	732.89	737.75	2048.08

## References

[1] Haupt P. (1999). On the Thermomechanical Modelling of Inelastic Material Behaviour. IUTAM Symposium on Micro- and Macrostructural Aspects of Thermoplasticity.

[2] Lemaitre J., Chaboche J.L. (1994). Mechanics of Solid Materials.

[3] Sandström R., Lagneborg R. (1975). A model for hot working occurring by recrystallization. Acta Metall..

[4] Roberts W., Ahlblom B. (1978). A nucleation criterion for dynamic recrystallization during hot working. Acta Metall..

[5] Sellars C.M. (1979). The Physical Metallurgy of Hot Working. Proceedings of the Conference on Hot Working and Forming Processes, Sheffield, England.

[6] Laasraoui A., Jonas J.J. (1991). Prediction of steel flow stresses at high temperatures and strain rates. MTA.

[7] Sommitsch C., Mitter W. (2006). On modelling of dynamic recrystallisation of fcc materials with low stacking fault energy. Acta Mater..

[8] Brown A.A., Bammann D.J. (2012). Validation of a model for static and dynamic recrystallization in metals. Int. J. Plast..

[9] Poliak E.I., Jonas J.J. (1996). A one-parameter approach to determining the critical conditions for the initiation of dynamic recrystallization. Acta Mater..

[10] Mejía I., Bedolla-Jacuinde A., Maldonado C., Cabrera J.M. (2011). Determination of the critical conditions for the initiation of dynamic recrystallization in boron microalloyed steels. Mater. Sci. Eng. A.

[11] Ebrahimi R., Solhjoo S. (2007). Characteristic points of stress-strain curve at high temperature. J. Southeast Univ..

[12] Gottstein G., Frommert M., Goerdeler M., Schäfer N. (2004). Prediction of the critical conditions for dynamic recrystallization in the austenitic steel 800H. Mater. Sci. Eng. A.

[13] Poliak E.I., Jonas J.J. (2003). Initiation of dynamic recrystallization in constant strain rate hot deformation. ISIJ Int..

[14] Yazdipour N., Davies C., Hodgson P.D. (2008). Microstructural modeling of dynamic recrystallization using irregular cellular automata. Comput. Mater. Sci..

[15] Zahiri S.H., Davies C.H., Hodgson P.D. (2005). A mechanical approach to quantify dynamic recrystallization in polycrystalline metals. Scr. Mater..

[16] Poliak E.I., Jonas J.J. (2003). Critical strain for dynamic recrystallization in variable strain rate hot deformation. ISIJ Int..

[17] Kim S.-I., Lee Y., Lee D.-L., Yoo Y.-C. (2003). Modeling of AGS and recrystallized fraction of microalloyed medium carbon steel during hot deformation. Mater. Sci. Eng. A.

[18] Bambach M. (2013). Conditions for consistent implementation of flow stress models incorporating dynamic recrystallization into finite element simulation codes. MSF.

[19] Bambach M. (2013). Implications from the Poliak–Jonas criterion for the construction of flow stress models incorporating dynamic recrystallization. Acta Mater..

[20] Kolmogorov A. (1937). On the statistical theory of the crystallization of metals. Bull. Acad. Sci. USSR Math. Ser..

[21] Johnson W.A., Mehl R. (1939). Reaction Kinetics in Processes of Nucleation and Growth. Trans. Metall. Soc. AIME.

[22] Avrami M. (1939). Kinetics of Phase Change. I General Theory. J. Chem. Phys..

[23] Cahn J.W. (1956). The kinetics of grain boundary nucleated reactions. Acta Metall..

[24] Speich G.R., Fisher R.M., Margolin H. (1966). Recrystallization in rapidly heated 3 1/4% silicon iron. Recrystallization, Grain Growth and Textures.

[25] Bambach M. (2016). Process and Materials Modeling in Metal Forming.

[26] Mecking H., Kocks U.F. (1979). A Mechanism for Static and Dynamic Recovery. Strength of Metals and Alloys.

[27] Estrin Y., Tóth L.S., Molinari A., Bréchet Y. (1998). A dislocation-based model for all hardening stages in large strain deformation. Acta Mater..

[28] Mecking H., Kocks U.F. (1981). Kinetics of flow and strain-hardening. Acta Metall..

[29] Yanagimoto J., Karhausen K., Brand A.J., Kopp R. (1998). Incremental Formulation for the Prediction of Flow Stress and Microstructural Change in Hot Forming. J. Manuf. Sci. Eng..

[30] Karhausen K., Kopp R. (1992). Model for integrated process and microstructure simulation in hot forming. Steel Res..

[31] Takaki T., Hisakuni Y., Hirouchi T., Yamanaka A., Tomita Y. (2009). Multi-phase-field simulations for dynamic recrystallization. Comput. Mater. Sci..

[32] Ding R., Guo Z.X. (2002). Microstructural modelling of dynamic recrystallisation using an extended cellular automaton approach. Comput. Mater. Sci..

[33] Lin J., Liu Y. (2003). A set of unified constitutive equations for modelling microstructure evolution in hot deformation. J. Mater. Process. Technol..

[34] Jonas J.J., Quelennec X., Jiang L., Martin É. (2009). The Avrami kinetics of dynamic recrystallization. Acta Mater..

[35] Beynon J.H., Sellars C.M. (1992). Modelling Microstructure and Its Effects during Multipass Hot Rolling. ISIJ Int..

[36] Cabrera J.M., Ponce J., Prado J.M. (2003). Modeling thermomechanical processing of austenite. J. Mater. Process. Technol..

[37] Grätz K., Miessen C., Gottstein G. (2014). Analysis of steady-state dynamic recrystallization. Acta Mater..

[38] Gottstein G. (2012). A Different View on Dynamic Recrystallization. Mater. Sci. Forum.

[39] Cao Y., Di H., Misra R. (2014). The impact of aging pre-treatment on the hot deformation behavior of alloy 800H at 750 °C. J. Nucl. Mater..

[40] Bergström Y. (1972). A dislocation model for the strain-ageing behaviour of steel. Mater. Sci. Eng..

[41] Lohmar J., Bambach M. (2013). Influence of Different Interpolation Techniques on the Determination of the Critical Conditions for the Onset of Dynamic Recrystallisation. MSF.

[42] Bambach M., Heppner S., Steinmetz D., Roters F. (2015). Assessing and ensuring parameter identifiability for a physically-based strain hardening model for twinning-induced plasticity. Mech. Mater..

